# A Case of Acute Motor Sensory Axonal Neuropathy: A Variant of Guillain-Barré Syndrome, with Possible Syndrome of Irreversible Lithium-Effectuated Neurotoxicity

**DOI:** 10.1155/2020/4683507

**Published:** 2020-04-20

**Authors:** David Y. Liu, Jessica R. Hollenbach, Jason A. Gregorin, Jonathan H. Wynbrandt

**Affiliations:** ^1^Internal Medicine, University Hospitals Regional Hospitals, Richmond Heights, OH 44143, USA; ^2^Lake Erie College of Osteopathic Medicine, Erie, OH 16509, USA

## Abstract

Acute Motor Sensory Axonal Neuropathy (AMSAN) is a rare and severe variant of Guillain-Barré syndrome (GBS) that has a prolonged recovery course. GBS is often suspected due to ascending muscle weakness, sensation difficulties, respiratory compromise, and antecedent diarrhea. The diagnosis of GBS is supported by cerebrospinal fluid analysis showing albuminocytologic dissociation. Electromyogram and nerve conduction study confirm the diagnosis and allow for further classification by variant. Treatment involves either IV immune globulins or plasmapheresis, and patients typically recover. However, depending on the variant and severity, patients may ultimately require prolonged mechanical ventilation with tracheostomy. In these cases, they may continue to have persistent muscle and sensation abnormalities requiring long-term care. We present a unique case of a 38-year-old female patient with decade-long use of lithium for bipolar disorder that presented with acute lithium toxicity. Though she was ultimately diagnosed with AMSAN, the Syndrome of Irreversible Lithium-Effectuated Neurotoxicity (SILENT) may have also contributed to her persistent neurological sequelae.

## 1. Background/Introduction

Guillain-Barré syndrome (GBS) is a set of acute immune-mediated peripheral polyneuropathies in response to antecedent, usually gastrointestinal, infection causing demyelination or axonal damage. It classically presents with progressive ascending weakness, decreased reflexes, and peripheral paresthesia. Altered mentation on presentation and chronic neurological sequelae greater than two months are atypical and may suggest another contributing diagnosis. We present an atypical case of GBS in which chronic use and acute toxicity of lithium may have caused the Syndrome of Irreversible Lithium-Effectuated Neurotoxicity (SILENT). This syndrome causes persistent neurological sequelae and may have contributed to the persistent motor weakness and altered sensation in a patient diagnosed with GBS.

GBS is a heterogeneous condition comprised of several variant forms that fall into two categories: demyelinating and axonal. The most common variant of GBS in the United States is Acute Inflammatory Demyelinating Polyneuropathy (AIDP). It presents with progressive, symmetric muscle weakness and decreased deep tendon reflexes and accounts for 85–90% of cases. Demyelination occurs at the level of the nerve root, and sensory nerves are preserved. Acute Motor Axonal Neuropathy (AMAN) is an axonal variant of GBS and accounts for 5–10% of cases. AMAN resembles AIDP as motor fibers are again affected, but deep tendon reflexes and sensation are typically preserved. It is distinguished from AIDP by axonal involvement on nerve conduction study. Acute Motor Sensory Axonal Neuropathy (AMSAN), a rare and severe variant of GBS, involves axonal degeneration of both sensory and motor fibers.

A diagnosis of GBS is supported by findings of albuminocytologic dissociation on lumbar puncture. This consists of an elevated cerebrospinal fluid (CSF) protein with a normal CSF white blood count. Albuminocytologic dissociation is present in 50–66% of cases in the first week of onset and >75% by third week of onset. Electromyography (EMG) and nerve conduction study (NCS) confirm the diagnosis and allow for further categorization by variant. Treatment includes plasmapheresis or IV immune globulins and supportive care [1–3].

SILENT is a rare complication of lithium toxicity. Lithium, first introduced in psychiatry in 1949, has long been known to have acute toxicity with a narrow therapeutic range. Symptoms of lithium toxicity include vomiting, diarrhea, cardiac arrhythmias, muscle weakness, tremor, ataxia, and delirium. Acute lithium toxicity is initially treated with aggressive fluid hydration, and hemodialysis may be required. In some rare cases, SILENT may develop despite adequate removal of lithium. While the mechanism for SILENT is not known, it has been suggested that CNS demyelination may occur and contribute to symptoms of cerebellar dysfunction, extrapyramidal symptoms, brainstem dysfunction, and dementia. Diagnosis is largely clinical based on neurological deficits caused by lithium in absence of prior neurological disease with neurological sequelae lasting longer than two months despite lithium cessation. Once irreversible damage sets in, treatment for SILENT is largely supportive care [4, 5].

## 2. Case Report

A 38-year-old Caucasian female with a medical history of bipolar disorder and anxiety presented to the emergency department at the recommendation of her psychiatrist due to concern for lithium toxicity after failing to correctly taper her lithium as instructed. She had no history of electro-convulsive therapy (ECT) and her medications at the time included use of lithium for greater than 10 years as well as lorazepam, sertraline, topiramate, and eszopiclone. Upon presentation, the patient was lethargic but was able to follow commands and responded to simple yes/no questions. She had poor short-term memory and was unable to elucidate many details of her medical history, including her medications. Her husband was present and provided the progression of symptoms. One month prior to presentation, the patient had diarrhea for one week that spontaneously resolved. Shortly after the resolution of her diarrheal illness, she developed weakness in the lower extremities that caused increasing difficulty with ambulation. One week prior to presentation, she began vomiting several times a day without significant abdominal discomfort and became increasingly lethargic. Additionally, the patient reported cold intolerance, loss of appetite, dry skin, and thinning hair for the past month.

The patient's vitals were stable and physical exam was unremarkable aside from an unsteady gait with no focal motor or sensory deficits. Labs were significant for a leukocytosis of 16,100/ml, decreased sodium of 130 mmol/L, decreased potassium of 2.7 mmol/L, with a creatinine that was at her baseline of 1.03 mg/dL. A head CT showed no acute process and chest X-ray showed possible atelectasis. Urinalysis indicated a urinary tract infection, growing *Escherichia coli* on reflex culture, and antibiotics were started. She had an elevated lithium level of 1.85 mmol/L (normal range 0.6–1.2 mmol/L) with an unknown baseline lithium level. Her TSH was elevated at 14.77 mlU/L with a low T4 0.68 ng/dL. Her lithium was discontinued, and she underwent aggressive IV fluid hydration, resulting in her lithium level decreasing to 0.36 mmol/L after 10 days. Hemodialysis was not required, and her mentation improved. She was placed on perphenazine and propranolol for anxiety and agitation, and started on levothyroxine for hypothyroidism. Her sertraline dose was decreased and planned to be discontinued in one week. However, her generalized weakness remained, which was attributed at the time to her continued symptomatic hypothyroidism and deconditioning. She was then discharged to a skilled nursing facility on perphenazine, propranolol, topiramate, and levothyroxine.

One week later, she returned to the emergency department with worsened generalized weakness and delirium. There were no changes to medications since discharge. She was extremely lethargic, unable to provide any history, and was only able to answer short yes/no questions. She also presented with a fever as well as tachycardia and tachypnea. Her ABG showed a pH of 7.49, pCO_2_ of 21 mmHg, and pO_2_ of 58 mmHg. The patient was placed on a nonrebreather mask at 15 L/min of oxygen. Physical exam demonstrated lung crackles on auscultation and flaccid muscle tone and sensory loss in all extremities. Labs were significant for an elevated lactate of 4.4 mmol/L, leukocytosis of 11,400/ml, normal sodium of 139 mmol/L, low potassium of 3.4 mmol/L, and a normal creatinine of 0.69 mg/dL. Troponin was 0.05 ng/dL with sinus tachycardia on EKG with no ST changes or QT prolongation; further 3 hour and 6 hour troponin showed no evidence of ischemia. Serum lithium level was undetectable and she had a normal TSH (3.64 mlU/L), T4 (1.43 ng/dL), and T3 (80 ng/dL). CT angiography was negative for pulmonary embolism, though it did reveal pneumonia. Broad spectrum antibiotics and IV fluids were started. CT abdomen and pelvis showed no acute findings. CT head and MRI were negative for ischemia or intracranial masses. EEG demonstrated slow waveforms indicative of metabolic encephalopathy. Lumbar puncture (LP) was performed, and CSF analysis showed an elevated protein of 142 mg/dL, glucose of 91 mg/dL, and total white blood cell count of 0. CNS infectious etiology was ruled out based on the results of the LP, but the albuminocytologic dissociation raised concern for GBS.

Her delirium slowly resolved with antibiotics by hospital day 4, but the patient's flaccid muscle weakness remained. She was unable to sit up unassisted, unable to grasp a cup handle, or push a button on the TV remote. Though the patient's oxygen demands were slowly titrated down from 15 L/min to 6 L/min, the patient progressively began to have difficulty breathing and she developed worsening hypoxia on hospital day 5, eventually leading to respiratory failure requiring mechanical ventilation. The diagnosis of GBS was then suspected due to findings of respiratory muscle failure, flaccid weakness in all extremities, sensory loss, and albuminocytologic dissociation in CSF. EMG/NCS confirmed axonal motor and sensory peripheral neuropathy consistent with AMSAN. Despite undergoing five rounds of plasmapheresis, the patient was unable to be weaned off mechanical ventilation and ultimately required chronic ventilation with tracheostomy as well as PEG tube placement. She continued to have persistent sensory and motor deficits and was discharged to long term rehabilitation six weeks after her initial admission. On chart review three months later, the patient had persistent neurological deficits that were unchanged from the time of discharge.

## 3. Discussion

Although a diagnosis of GBS was confirmed via albuminocytologic dissociation on LP and AMSAN on EMG/NCS, the patient continued to have persistent neurological sequelae of muscle weakness and decreased sensation in all extremities after completion of treatment with plasmapheresis. This atypical persistence of symptoms along with the initial presentation of altered mental status, raised the question of an alternative, or additional diagnosis, such as SILENT.

Though GBS and SILENT both present with acute neurological symptoms, deficits in muscle strength in GBS usually resolve but persist in SILENT. Rajabally and Uncini found that >80% of all GBS cases were able to walk independently at six months, while only 14% were left with a severe disability [6]. However, a three variable prognostic scoring system developed by van Koningsveld et al. helps further distinguish the likelihood of prolonged or permanent deficits. Although the age of onset for the patient was low at 18–40 years old (0 points), she had preceding diarrhea (1 point) and ultimately required mechanical ventilation (5 points). A total of 6 out of 7 possible points were found to correlate to a 55% risk of inability to walk after admission [7]. This poor prognosis, in addition to our patient having the more severe and rarer AMSAN variant of GBS, led to a greater likelihood of chronic neurological sequelae.

Our patient's delirium on initial presentation does seem to be more consistent with a diagnosis of SILENT rather than GBS. Our patient's improving mentation coincided with normalization of lithium levels on her first visit, though improvement was slow. However, even if lithium was maintained in therapeutic range, neurological toxicity may still have insidiously developed [5, 8]. Symptomatic hypothyroidism may have also contributed to this presentation, as it also causes cognitive impairment with slowed mentation, poor concentration, and decreased short-term memory [9]. While her presentation of delirium on her second visit occurred alongside undetectable lithium levels and normal TSH, T4, and T3 levels, this is likely attributed to sepsis secondary to pneumonia and improved expectantly with antibiotic therapy. While the patient did have initial delirium and persistent deficits more consistent with SILENT, the patient did not have continued dementia as is typical for SILENT.

This case exemplifies the importance of keeping a broad differential when presented with generalized weakness and delirium. As the patient had documented lithium toxicity and hypothyroidism, all focus was on aggressive IV fluid hydration and thyroid replacement with the expectation that generalized weakness would gradually improve over time. However, with readmission for worsened weakness, further workup led to the diagnosis of GBS. Although we can find no precedent of lithium toxicity and symptomatic hypothyroidism masking GBS, there are other cases that show the difficulty in diagnosing GBS when other diagnoses are more evident. Jung et al. demonstrated a female that had rapidly progressive motor weakness with a history of MRI confirmed lumbar spinal stenosis that did not improve despite surgical decompression [10]. Sarada and Sundararajan exhibited a patient with confirmed diagnosis of pulmonary sarcoidosis that was readmitted 2 weeks later for acute progressive distal to proximal limb weakness that was initially misdiagnosed as neurosarcoidosis [11].

In conclusion, while our patient did not improve tremendously with plasmapheresis, as is expected in GBS, we believe she had a greater chance of long-term disability because of the more severe variant of AMSAN and her poor prognostic score. Because of her decade long use of lithium and lithium toxicity at initial presentation, it is, however, a distinct possibility that SILENT may have also been a contributing factor to her persistent neurological sequelae, despite not having the chronic mental status changes typically seen in SILENT.
